# Clinical management of sepsis-associated acute respiratory distress syndrome: current evidence and future directions

**DOI:** 10.3389/fmed.2025.1531275

**Published:** 2025-05-26

**Authors:** Liang Liu, Linguangjin Wu, Ying Chen, Rou Deng, Yingying Hu, Yanjie Tu, Bangjiang Fang

**Affiliations:** ^1^Department of Emergency, Longhua Hospital, Shanghai University of Traditional Chinese Medicine, Shanghai, China; ^2^Department of Febrile Disease, School of Traditional Chinese Medicine, Shanghai University of Traditional Chinese Medicine, Shanghai, China; ^3^Department of Emergency, The First Affiliated Hospital of Henan University of Science and Technology, Luoyang, Henan, China; ^4^Institute of Critical Care, Shanghai University of Traditional Chinese Medicine, Shanghai, China

**Keywords:** sepsis-associated acute respiratory distress syndrome (SA-ARDS), inflammatory response, pathomechanisms, subphenotype, clinical management

## Abstract

Sepsis is a life-threatening condition characterized by organ dysfunction resulting from a dysregulated host response to infection. The lungs are among the first and most significantly affected organs in sepsis. Pulmonary infections or systemic inflammatory cascades triggered by various pathogens can lead to acute and diffuse pulmonary damage, often manifesting as persistent hypoxemia. The COVID-19 pandemic has highlighted critical knowledge gaps in SA-ARDS management, necessitating paradigm reevaluation under the new global definition of ARDS. This paper analyzes the pathomechanisms and subphenotype characteristics of SA-ARDS, reviews recent advances in clinical management, such as fluid resuscitation, antimicrobial therapy, immune modulation, respiratory support, microcirculatory improvement, and traditional Chinese medicine (TCM) therapies, and addresses controversial issues and areas requiring further investigation.

## 1 Introduction

Sepsis is a prevalent complication associated with severe infections, major trauma (burns), shock, and surgical procedures. As a syndrome characterized by high morbidity, mortality, healthcare cost, and poor recovery outcomes, sepsis represents a significant threat to human health (1). Statistics indicate that approximately 48.9 million new cases of sepsis occur annually, with nearly 11 million sepsis-related deaths worldwide, accounting for 19.7% of the deaths from various causes (2). The average median hospitalization cost per patient throughout the course of life-saving treatment can reach $32,421, placing a considerable burden on healthcare systems (3). Even among survivors discharged from the hospital, 12.2% are at risk of unplanned rehospitalization within 30 days, 44.2% may succumb within 1 year, and 16.7% experience long-term physical disability or cognitive impairment, resulting in a prognosis worse than for other diseases (4–6). In recognition of this crisis, the World Health Organization (WHO) officially identified “improving the prevention, diagnosis and clinical management of sepsis” as one of the major tasks in 2017, urging governments worldwide to intensify their efforts to address this urgent issue (7).

The lungs are among first and most significantly affected target organs in sepsis. Respiratory infections or systemic inflammatory responses triggered by various pathogens can result in acute, diffuse, inflammatory lung injury, and acute respiratory failure. The primary clinical manifestations are refractory hypoxemia and bilateral diffuse opacities on imaging. It is estimated that approximately 25–50% of sepsis patients develop acute respiratory distress syndrome (ARDS). The occurrence of ARDS has been independently correlated with increased mortality intensive care unit (ICU), prolonged hospitalization, and reduced ventilator-free days in sepsis patients (8–10). As the leading cause of ARDS, sepsis is closely linked to an ICU mortality rate of 35–46% among ARDS patients. In recent years, pandemics such as influenza and COVID-19 have contributed to a marked rise in the incidence of sepsis-associated acute respiratory distress syndrome (SA-ARDS), further escalating the need for respiratory support and the risk of mortality (11).

In 2021, the new global definition of ARDS established in response to the COVID-19 pandemic, broadening the diagnostic criteria to include partial acute hypoxic respiratory failure (AHRF) outside the scope of the original Berlin definition (12). This revision facilitated the more timely recognition and treatment of SA-ARDS. However, controversy persists among researchers regarding the most effective therapeutic approaches, leading to a lack of consensus on a definitive treatment protocol or standardized clinical procedures (1, 13). This paper aims to summarize recent advances in the management of SA-ARDS, focusing on anti-infection, anti-inflammatory, immune regulation, respiratory support, and other therapies. Additionally, it explores the heterogeneity in treatment response across different SA-ARDS subphenotypes, with the goal of informing the development of personalized, precision-based treatment strategies.

## 2 Pathomechanisms

The pathomechanism of SA-ARDS is multifactorial, involving complex interactions between inflammatory injury, immune dysregulation, coagulation disturbances, and their respective signaling pathways. Pathogen invasion into the lungs (14, 15) or a systemic inflammatory response resulting from extrapulmonary infections (16) triggers antigen recognition, presentation, and immune activation, thereby initiating inflammatory signaling pathways (17). Large amounts of inflammatory mediators infiltrate the lungs, including interleukin (IL)-1β, IL-6, tumor necrosis factor (TNF)-α, chemokines, granulocyte macrophage colony-stimulating factor (GM-CSF), and intercellular adhesion molecule (ICAM)-1, which promote recruitment of immune cells in the lungs and an uncontrolled inflammatory cascade (18). Activated neutrophils and inflammatory factors can contribute to the necrosis of alveolar epithelial and vascular endothelial cells, accompanied by disruptions in alveolar surfactants. These events lead to increased permeability of pulmonary epithelium and vascular endothelium, resulting in leakage of proteins and cellular contents, thereby causing alveolar and interstitial edema and amplifying pro-inflammatory signals (9, 19–21). Concurrently, activated alveolar macrophages and multinucleated leukocytes release a large number of reactive oxygen species and oxidized molecules. Oxidative stress results in lipid peroxidation of cell membranes and the accumulation of oxidized proteins, further exacerbating the apoptosis of alveolar cells and the disruption of lung epithelium (8, 22, 23).

Damage to and activation of vascular endothelial cells lead to the exposure of coagulation factors on the endothelial surface. At the same time, leukocytes (including neutrophils, monocytes, and eosinophils) release microvesicles and neutrophils extracellular traps (NETs), that activate procoagulant substances such as tissue factors and platelet-activating factors, thereby initiating the exogenous coagulation cascade. This process promotes the activation, migration, and recruitment of platelets to the injured site to form microvascular thrombosis. Increased pulmonary vascular dead space (pulmonary microcirculation thrombosis) is associated with a poor prognosis in SA-ARDS. The production and activation of intravascular thrombin further amplify inflammatory signaling pathways and promote the aggregation of inflammatory cells through multiple mechanisms. This positive feedback loop between coagulation and inflammation ultimately culminates in disseminated intravascular coagulation (DIC) and septic shock (24–28).

**FIGURE 1:**
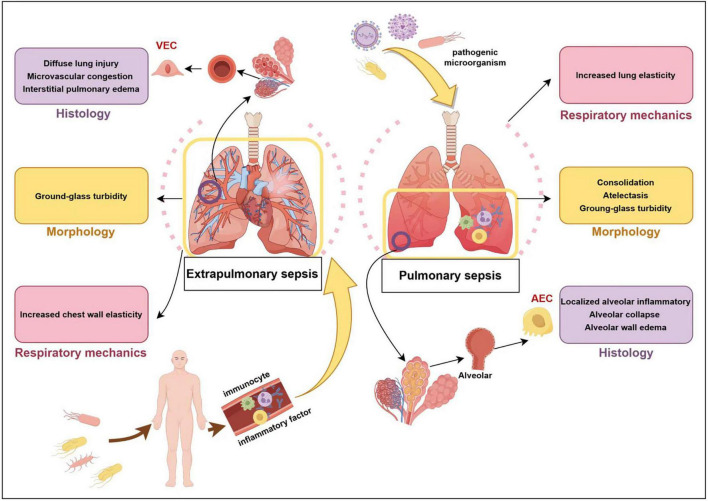
Extrapulmonary sepsis vs. pulmonary sepsis. VEC, vascular endothelial cell; AEC, alveolar epithelial cell.

The aforementioned pathological processes can cause a series of clinical manifestations. Alveola-capillary barrier injury result in interstitial and alveolar edema, reduced lung volume, increased lung elasticity, decreased compliance, and elevated respiratory work (13). Inactivation or decreased production of alveolar surfactants leads to reduced alveolar surface tension and alveolar collapse, which is characterized by ventilation obstruction and a good response to positive end-expiratory pressure (PEEP) (29). Diffuse alveolar filling leads to a severe imbalance in the ventilation/blood flow ratio, pulmonary diffusion dysfunction, bilateral diffuse shadowing on imaging, and hypoxemia. Pulmonary vascular endothelial injury and microvascular thrombosis contribute to increased dead space ventilation and pulmonary hypertension, with clinical manifestations of high minute ventilation, hypercapnia, and right heart failure. The systemic inflammatory response can induce multiple organ dysfunction, potentially progressing to shock and death (9). Based on the infection source, inflammatory response, and morphological features, SA-ARDS has been classified into various subphenotypes. There are differences between SA-ARDS caused by pulmonary infections and that resulting from extrapulmonary infections, as shown in Figure 1 (30–34). However, no obvious distinctions were noted regarding the duration of mechanical ventilation (MV), length of hospitalization, or 28-day mortality rate (35, 36).

## 3 Fluid management

Fluid management in SA-ARDS involves two opposing risks: circulatory failure and fluid overload. Aggressive bundle therapy is the cornerstone of effective sepsis treatment, with intravenous infusion of crystalloids ≥ 30 mL/kg within the first 3 h is often recommended (1, 37). However, studies have shown that rehydration exceeding 5 L within 24 h of ICU admission may increase mortality risk (38). This is mainly due to extensive damage to the patients’ capillary barriers and the increase in total circulating blood volume, leading to enhanced tissue leakage, elevated pulmonary hydrostatic pressure, and the development of pulmonary edema (39), which is independently correlated with adverse outcomes (such as prolonged duration of MV, extended ICU stay, and higher mortality) (40, 41). Ingelse et al. explored a restrictive fluid strategy in the early stage of ARDS but found it insufficient to limit pulmonary edema, while also leading to increased use of vasoactive drugs without any direct benefit to cardiopulmonary function (42). The FACTT study revealed an interaction between SA-ARDS subphenotypes and fluid management strategies. The hyperinflammatory subphenotype, characterized by significantly elevated serum levels of IL-8, tumor necrosis factor receptor-1 (TNFr1), etc., and decreased bicarbonate levels, required more vasopressor support, and exhibited a lower 90-day mortality rate when randomly assigned to a fluid-conservative strategy compared to the fluid-liberal group (40% vs. 50%) (43).

In the fluid management debate, the concepts of adequate initial fluid resuscitation (AIFR) and conservative late fluid management (CLFM) have been introduced. AIFR was defined as administering an initial fluid bolus of ≥ 20 mL/kg before and achieving a central venous pressure of ≥ 8 mmHg within 6 h after the onset of therapy with vasopressors. CLFM referred to maintaining even-to-negative fluid balance, as measured over at least 2 consecutive days during the first 7 days after septic shock onset. It was found that the in-hospital mortality was significantly lower in patients receiving both AIFR and CLFM compared to those with AIFR only, CLFM only, or neither (18.3% vs. 41.9% vs. 56.6% vs. 77.1%, *p* < 0.01). This suggests that a staged fluid management strategy can effectively shorten the duration of ventilator use and ICU stay in SA-ARDS patients. The combined approach of early fluid resuscitation and late conservative fluid rehydration may represent a more optimal strategy for SA-ARDS (44, 45). According to the four phases of fluid management: resuscitation, optimization, stabilization, and evacuation (46), the early resuscitation phase based on the principle of 1 h bundle therapy, aiming to fully improve tissue perfusion and avoid ischemia and necrosis of the important organs. The fluid resuscitation volume is typically controlled to 2.5–5.0 L (47). In the later optimization phases, hemodynamic monitoring is used to assess each patient’s volume status and fluid reactivity, allowing for dynamic and precise adjustments in the type and volume of fluids to prevent overload, which could exacerbate heart failure and pulmonary edema.

## 4 Timing of antimicrobial therapy

Infection during SA-ARDS comes from a wide range of sources, with Gram-positive or Gram-negative bacterial infections being the most common, followed by viral or fungal infections. However, the incidence of viral sepsis has surged dramatically during viral pandemics, such as those caused by *SARS-CoV-2*, *Alphainfluenzavirus*, *Betainfluenzavirus, Respiratory syncytial viruses*, and *Noroviruses* (48). Viral infections tend to overlap with bacterial infections due to physical barrier disruption, host immunosuppression, invasive manipulation, and virus-bacteria interactions (49). Mixed viral-bacterial infections can account for 22.5–65.0% of sepsis cases, and are strongly associated with pathogen resistance and increased mortality risk (50–53). The rate of infections with these pathogens increases during acute viral infection, as reported during the COVID-19 pandemic (54), community-acquired infections are most common with *Staphylococcus aureus*, *Streptococcus pneumoniae*, and *Klebsiella pneumoniae* (50, 51). Hospital secondary infections more frequently involve *Pseudomonas aeruginosa*, *Klebsiella genus*, *Enterobacteriaceae genus*, methicillin-resistant *Staphylococcus aureus*, and *Enterococcus genus* (51, 52, 54). Given the critical nature of SA-ARDS, the initiation of broad-spectrum antibiotics is recommended at the earliest possible stage, even when clear pathogenetic evidence of bacterial infection is absent, especially in patients with prolonged prehospital delays, coexisting immune disorders, invasive interventions, and ongoing corticosteroid therapy. Serum procalcitonin (PCT) and C-reactive protein (CRP) may aid in bacterial diagnosis, with PCT demonstrating negative predictive values ranging from approximately 91.1–94.0%. When PCT levels exceed 0.05 μg/L, it is difficult to exclude the possibility of mixed infection (55, 56).

Although both delayed recognition and delayed administration of antibiotics significantly increase in-hospital mortality (57), the optimal duration of administration remains uncertain. A systematic review of 35 clinical studies found that two-thirds of the studies reported an association between the duration of antibiotic use and mortality. However, the time-to-administration measures varied widely across studies, with the first dose ranging from 1 to 6 h. Some studies suggested no significant survival benefit for sepsis patients who received antibiotics early. Since most of the studies analyzed suspected sepsis and septic shock in a mixed cohort, researchers have proposed that the risk trade-off for delaying treatment primarily involves the progression to shock (58). Subgroup stratification based on the timing of antibiotic initiation in SA-ARDS patients is necessary. After adjusting for factors such as disease severity and maximum time-to-antibiotics, it was found that each 1-h delay in treatment was significantly associated with an increase in septic shock mortality. However, only delays greater than 6 h were associated with higher mortality in patients with non-shock sepsis (59). In a real-world study involving 166,559 patients, individuals were grouped based on probable/possible sepsis and shock/no shock using a 2*2 table. The results showed that in the probable sepsis group, the median time to antibiotic administration was 2.7 h in the shock subgroup and 3.2 h in the non-shock subgroup. In the possible group, the median time was 6.9 h in the shock subgroup and 5.5 h in the non-shock subgroup. Hospitalization mortality was significantly lower in patients without shock compared to those with shock (2–3% vs. 12–17%) (60). Therefore, strict time-orientation of 1 or 3 h in suspected sepsis patients without shock may lead to misdiagnosis and unnecessary antibiotic use. Refining the classification of SA-ARDS may provide more flexibility in timing the first dose of antibiotics in suspected sepsis patients, allowing for time to gather evidence.

Antibiotic use should be promptly adjusted and adequately evaluated for de-escalation once pathogen identification and drug sensitivity results are available. Prolonged antibiotic treatment may increase the risk of drug resistance, *Clostridium difficile* infection, antibiotic-related toxicity, and healthcare costs. In a study by Chastre et al., patients were treated with antibiotics for either 8 or 15 days. They found that, aside from patients with non-fermentative Gram-negative bacterial infections (including *Pseudomonas aeruginosa*), there was no significant difference in recurrence rate of pulmonary infection, MV duration, organ failure days, ICU stays, or mortality between the 8-day treatment and the 15-day treatment group. Furthermore, the occurrence of multi-drug resistant organisms (MDRO) was lower in the 8-day group (61). These findings led to the development of the “shorter is better” approach to anti-infective therapy. However, Busch et al. noted that most current studies on antibiotic duration are non-inferiority tests, and nearly 80% of sepsis patients may have hidden lesions due to immunosuppression, making it challenging to fully apply this approach in the complex and dynamic management of sepsis patients (62).

Research have found that serum PCT levels correlated positively with infection severity, and appropriate antibiotic treatment can reduce PCT levels (63). Guiding the initiation, continuation, discontinuation, and replacement of antibiotics based on PCT levels has been shown to improve survival and shorten treatment duration (64). In the PROGRESS trial, Discontinuation was defined as a reduction in PCT levels by ≥ 80% from baseline or a PCT concentration ≤ 0.5 μg/L by day 5. The results demonstrated that, compared to the standard care, the PCT-guided discontinuation group not only reduced the median duration of antibiotic treatment from 10 to 5 days, but also decreased 28-day mortality and hospitalization costs. More importantly, it significantly reduced the incidence of infective adverse events at 180 days (new case of MDRO infection, new case of *Clostridium difficile* infection, and death associated with MDROs or *Clostridium difficile* baseline infections), and positively affected both short- and long-term outcomes in sepsis patients (65).

## 5 Anti-inflammatory

### 5.1 Corticosteroids

The inflammatory response is a key link in the onset and progression of SA-ARDS, and anti-inflammatory drugs play a moderately important role in improving lung physiology and mitigating poor prognosis. Among these, corticosteroids such as methylprednisolone, hydrocortisone, dexamethasone, and fludrocortisone are commonly used due to their extensive anti-inflammatory and immunomodulatory effects (66). Compared to conventional treatments, patients combined with dexamethasone treatment exhibit significantly shorter duration of MV, improved sequential organ failure assessment (SOFA) scores, and substantial reductions in the levels of inflammatory factors such as IL-6 in the blood and lungs (67, 68). However, a synthesis of several clinical trials has shown that while corticosteroids are important for reducing organ dysfunction and reversing shock in patients with septic shock requiring vasoactive agents, there is no definitive recommendation for their use in non-shock sepsis or SA-ARDS (1, 69). In a double-blind randomized controlled trial, it was found that patients received 50 mg of hydrocortisone every 6 h showed improvements in lung physiology compared with the placebo group, but there were no significant differences in acute physiology and chronic health evaluation (APACHE) II scores or 28-day mortality. It suggested that significant physiological improvement with corticosteroids in SA-ARDS does not necessarily translate into an overall survival benefit (70). In contrast, a study on sepsis due to community-acquired pneumonia (CAP) in which 648 patients had comorbid ARDS, showed that hydrocortisone plus fludrocortisone treatment significantly reduced mortality compared with placebo (71). Despite the lack of support from clinical studies, some researchers have suggested that patients with SA-ARDS resulting from respiratory system infections may derive more benefit from corticosteroid treatment (72). Multiple steroid administration strategies should be tailored to the clinical context, and if necessary, the heterogeneity of corticosteroids effects in ARDS, depending on phenotypes and genotypes, should be explored by integrating precision medicine approaches (66, 73).

### 5.2 Statins

Statins, as inhibitors of 3-hydroxy-3-methylglutaryl-CoA reductase (HMGCR), can exert beneficial effects in severe infections through mechanisms such as anti-inflammatory, antioxidant, and anticoagulant actions. However, low cholesterol levels may impair immunity function, alter hormone levels, and reduce membrane receptor sensitivity, potentially increasing mortality in critical diseases. This has led to controversy surrounding the clinical application of statins in sepsis and ARDS (74–76). Although previous studies have concluded that statin use does not prevent onset of new organ failure or reduce 30-day all-cause mortality in SA-ARDS patients (77, 78), the effects of different statins vary. Specifically, simvastatin and atorvastatin have been shown to significantly improve the 30-day survival rate in sepsis patients, while rosuvastatin did not demonstrate a similar benefit (79). Recent secondary analyses of randomized controlled clinical trials (RCT), including the HARP-2 trial for simvastatin and the SAILS trial for rosuvastatin, found that the mortality of ARDS patients with high inflammatory subphenotypes was significantly improved and showed survival benefits at 28 days after simvastatin treatment. In contrast, no benefits were observed with rosuvastatin, especially in patients with low inflammatory subphenotypes. This differential response may be attributed to simvastatin’s ability to reduce the secretion and activation of inflammasomes, such as IL-18 and IL-1β (80–82). Statins like rosuvastatin are hydrophilic, while simvastatin and atorvastatin are lipophilic. Whether statins improve the outcome of sepsis and ARDS may depend on their hydrophilic or lipophilic properties. In support of this, Wang et al. confirmed that lipophilic statins, such as simvastatin and mevastatin, can inhibit the activation of inflammasome (83). Genome-wide association studies (GWAS) also revealed that the expression of HMGCR is closely related to elevated circulating inflammatory factors, such as IL-18 and C-C motif chemokine ligand 2 (CCL2). Statins may mitigate the excessive inflammatory response in SA-ARDS by targeting HMGCR (84). Therefore, future clinical studies evaluating the safety and efficacy of statin therapy for SA-ARDS should consider the inflammatory subphenotype and pharmacochemical properties of the drugs.

## 6 Immune regulation

In the acute phase of SA-ARDS, systemic inflammatory response syndrome (SIRS) driven by a cytokine storm is the predominant manifestation. Overactivated immune cells and pro-inflammatory factors not only fail to effectively eradicate pathogens, but also contribute to severe cell death, tissue damage and organ dysfunction (85). Therefore, early identification and timely, targeted intervention to block or eliminate the excessive inflammatory response are crucial. Inhibiting pro-inflammatory cytokine activity is a fundamental strategy to prevent uncontrolled inflammation. For instance, in SA-ARDS patients with elevated levels of CRP and IL-6, the application of tocilizumab (a humanized monoclonal antibody targeting the IL-6 receptor) has been shown to effectively reduce the probability of intubation and decrease mortality risk in ARDS patients (86, 87). Complement activation was reported to play a key role in driving the inflammatory response in patients suffering from ARDS associated with acute SARS-CoV-2 infection. Targeting complement dysregulation has proven to be an effective strategy for mitigating these effects (88, 89). Clinical trials evaluating complement inhibitors such as Narsoplimab and Eculizumab have demonstrated significant reductions in inflammatory markers and improved clinical outcomes in these patients (90, 91).

Due to cytokine consumption and inflammation damage, most patients with SA-ARDS rapidly progress to an immunosuppressed state, often leading to secondary infections that contribute to 70% approximately of sepsis-related death. Consequently, the research focus has shifted in the current clinical paradigm toward “immune stimulation therapy” aimed at countering immunosuppression (85, 92). Direct supplementation of inflammatory cytokines or cytokines with immune-stimulatory properties represents a potent strategy to enhance host immunity. For example, studies have shown that administration of CYT107 (a glycosylated recombinant human IL-7) via intramuscular injection significantly increases the absolute lymphocyte count, as well as circulating CD4 + and CD8 + T cells, by 3–4 times, with these effects sustained for several weeks. This intervention effectively reverses sepsis-induced depletion of immune effector cells and reduces the need of organ support (93, 94). Additionally, interferon (IFN)-β has been shown to promote the resolution of pulmonary inflammation and reduce pulmonary vascular leakage by blocking TNF-α/IL-10-mediated alveolar macrophage injury and enhancing alveolar neutrophil recruitment (95–97).

Immune checkpoint blockers (ICBs) represent a promising strategy for promoting the restoration of immune homeostasis. By blocking the negative co-stimulation pathways mediated by classical immune checkpoint receptors expressed on T-lymphocytes [including Programmed death-1 (PD1), cytotoxic T lymphocyte-associated protein 4 (CTLA-4), B and T lymphocyte attenuator (BTLA)], ICBs can reverse lymphocyte apoptosis and monocyte dysfunction, thus improving the risk of secondary infections and enhancing survival (98–101). Treatment with BMS-936559 (anti-PD-L1 antibody) and nivolumab (anti-PD-1 antibody) has been shown to significantly increase absolute lymphocyte counts and monocyte human leukocyte antigen (HLA)-DR over time, restoring immune function without triggering a cytokine storm. These findings provide strong evidence supporting the clinical safety, tolerability, and pharmacokinetics of ICBs (102–104). Additionally, mathematical models have suggested that when antibiotics alone are insufficient and the initial pathogen load is not excessively high, early combination therapy with 6 mg/kg of nivolumab with meropenem may help clear pathogens, reverse immunosuppression, and improve sepsis prognosis (105, 106).

## 7 Respiratory support

Non-invasive respiratory support including high-flow nasal cannula (HFNC) and non-invasive ventilation (NIV), is commonly employed as the first-line treatment in the management of SA-ARDS. These interventions help improve oxygenation, maintain physiologic airway protection, prevent diaphragmatic dysfunction, and reduce the risk of sedation and analgesia, airway opening, and respiratory muscle strain (1, 107).

When compared to various modes of respiratory support before intubation, the continuous positive airway pressure (CPAP)/NIV group demonstrated a significantly lower requirement for tracheal intubation and a reduced 30-day mortality rate compared to the conventional oxygen therapy (COT) group (108). While the likelihood of requiring tracheal intubation in the HFNC group was significantly lower than in the COT group, no significant difference was observed in 28-day mortality between the two groups. This indicated that non-invasive respiratory support offers distinct advantages over COT in reducing the risk of intubation, while further research is needed to address its impact on mortality (109–111). Although HFNC is currently the most widely used non-invasive ventilation modality (112), it is indistinguishable from NIV in terms of ventilation duration, escalation rate of respiratory support, or in-hospital mortality (113–115). This may be attributed to the interaction between oxygenation index (partial pressure arterial oxygen/faction of inspiration O_2_, PaO_2_/FiO_2_) and tidal volume. Due to PEEP and additional pressure-supporting effect, SA-ARDS patients with lower PaO_2_/FiO_2_ ratios are more likely to suffer from ventilate-induced lung injury (VILI) during NIV treatment, primarily related to high tidal volumes driven by respiratory effort (116). Therefore, patients with lower PaO2/FiO2 ratios may benefit more from HFNC in clinical practice, and HFNC is better tolerated and more comfortable. NIV is currently preferred in SA-ARDS patients with mixed respiratory failure or hypercapnia (117, 118).

When the condition is severe, non-invasive respiratory support may struggle to balance the benefits of avoiding sedation and intubation with the risks of patient self-inflicted lung injury (PSILI) and delayed intubation. In such cases, invasive mechanical ventilation (IMV) should be promptly considered. The respiratory oxygenation index [ROX, calculated as peripheral capillary oxygen saturation (SpO_2_)/FiO_2_/respiratory rate (RR)] and mROX (calculated as PaO_2_/FiO_2_/RR) can be used to predict the outcomes of HFNC. When the ROX is below 5.33 (2–6 h after HFNO initiation), 3.69 (6–12 h), and 6.07 (12–24 h), it is necessary to actively prepare for IMV (119, 120). In addition, who transition from CPAP to IMV exhibit higher respiratory rates, minute ventilation, tidal pleural pressures, mechanical power ratios, and lower alveolar-to-inhaled partial pressure (121). Numerous studies have demonstrated that SOFA score, simplified acute Physiological score II (SAPS II), SpO_2_, respiratory effort signals, and imaging manifestations of lung injury may more accurately predict the timing of IMV conversion compared to the ROX index. This may be related to silent hypoxia and oxygen flow settings (122, 123). Therefore, further research is needed to identify indicators for the success of non-invasive respiratory support. Currently, clinical monitoring remains essential, with comprehensive judgment based on a combination of respiratory mechanical indicators and overall systemic status.

## 8 Lung protective ventilation strategy

In SA-ARDS patients, the lung tissues typically exhibit normal areas, collapse regions, consolidation, inflammatory infiltration, and other pathological changes, with only a small part of the lung maintaining respiratory function. During positive pressure ventilation, collapsed alveoli may undergo repeated cycles of opening and closing, normal alveoli may become overexpanded, infiltrated lung regions may experience impaired ventilation, and consolidated areas may fail to recruit. VILI results from excessive alveolar deformation and cyclic opening and collapse. The lung protective ventilation strategy aims to minimize the risk of VILI while optimizing lung inflation by promoting more uniform ventilation across the lung (124).

Low tidal volume (4–8 mL/kg of predicted body weight) and lower inspiratory pressures (plateau pressure <30 cmH_2_O) are generally accepted in clinical practice to prevent excessive lung stretch and reduce inflammatory mediators release (1, 13, 125). However, there is ongoing debate regarding the optimal level of PEEP. Adequate PEEP can help open collapsed lung tissue to ensure oxygenation, but over-inflation may lead to lung damage and circulatory compromise. Data from the ALVEOLI, LOVS, and EXPRESS trials indicate no significant difference in mortality between the high-PEEP group (PEEP > 15 cmH_2_O or the maximum PEEP during lung recruitment) and the low-PEEP group, with similar incidences of pneumothorax and rates of vasopressors use. Nonetheless, high PEEP may improve lung function, shorten the duration of MV, prevent life-threatening hypoxemia, and reduce the need for rescue treatment (126–129). Subgroup analyses revealed heterogeneity in the therapeutic response of different ARDS subphenotypes to PEEP strategies. The hypoinflammatory subphenotype is more susceptible to alveolar injury at higher PEEP levels, which can increase mortality risk, whereas the hyperinflammatory subphenotype appears to derive greater benefit from higher PEEP (130, 131). Guidelines also emphasize the uncertainty surrounding the role of PEEP in SA-ARDS management (13). As a result, the optimal PEEP is often individualized, and needs to take full account of lung expansibility (the proportion of lung tissue restored by ventilation when airway pressure increases). In patients with low lung expansibility, high PEEP may only increase the inflation of the already open lung region, thus increasing the pressure and strain on the lungs, potentially leading to VILI, rather doing more harm than good (132). Computed tomography (CT) have shown that patients with low P/F ratios, multiple atelectatic areas, and poor lung compliance exhibit higher lung recruitability. In these cases, increasing PEEP from 5 to 15 cmH_2_O can significantly improve lung ventilation and PaO2 (133). Esophageal pressure monitoring, CT image, lung and diaphragm ultrasound, and electrical impedance tomography are commonly used to assess lung reperfusion and optimize PEEP settings in patients with SA-ARDS (134–138).

Prone position ventilation (PPV), which facilitates more even expansion of collapsed dorsal lung tissue, has gained attention during the COVID-19 pandemic, and promoted the widespread use of awake prone position (139). Early application of PPV has been shown to significantly enhance pulmonary recruitment in ARDS patients, reduce alveolar instability and hyperinflation observed under high PEEP, improve the overall ventilation/perfusion ratio, and decrease 28- and 90-day mortality (140, 141). However, when assessed using PPV responsiveness criteria (defined as a P/F ratio increase ≥ 20% after PPV indicating a good response), some investigators have found that not all ARDS patients can be adapted to PPV, with an overall non-response rate of approximately 32.6%. The effectiveness of PPV is independently correlated with the severity of lung injury, cardiac insufficiency, and hemoglobin concentration (142). Additionally, different lung lesion morphologies show variable responses to PPV. In SA-ARDS with predominantly subpulmonary solid lesions (focal subphenotype), which is characterized by significantly reduced dorsal ventilation and ventral alveolar hyperinflation, PPV can redistribute gravity-dependent zones, alleviate localized compression, reopen collapsed alveoli, and improve pulmonary ventilation and oxygenation. In contrast, patients with diffuse ventilation loss (non-focal subphenotype), where PPV action is limited due to extensive and uneven alveolar injury, tend to respond better to PEEP, which promotes alveolar reopening through more uniform pressure transfer (9).

## 9 Anti-microvascular thrombosis

In SA-ARDS, a “vicious cycle” is formed between coagulation and inflammation. Coagulation factor activation and platelet aggregation not only lead to fibrin deposition and microvascular thrombosis, but also trigger a pro-inflammatory response (27). As a result, platelet activation, migration and accumulation in the alveoli are the main features of SA-ARDS (28). Heparin, the most commonly used drug for managing coagulation disorders in the ICU, possesses anticoagulant, anti-inflammatory, anti-complement activities, and protease-modulating properties. Early prophylactic administration of heparin (subcutaneous injection within 48 h of ICU admission) improves Murray lung injury score, reduces the need for tracheotomy, and increases survival in patients with SA-ARDS. Its mechanism of action is related to the inhibition of inflammation-induced microvascular occlusion and hyaline membrane formation in the alveoli (143, 144).

Aspirin is thought to modulate the progression of sepsis and ARDS by inhibiting cyclooxygenase and nuclear factor kappa-B (NF-κB), while promoting the production of nitric oxide and lipoxins (28). In a lipopolysaccharide (LPS)-induced human model of SA-ARDS, any dose of aspirin was found to suppress pulmonary neutrophilic inflammation (145). Although several observational studies have indicated that pre-hospital, in-hospital, or long-term aspirin administration reduces mortality in patients with sepsis or ARDS, the results have shown considerable heterogeneity (146, 147). However, recent large-scale prospective randomized controlled trials (e.g., ANTISEPSIS trial and LIPS-A trial) failed to provide evidence the use of early aspirin prophylaxis in improving sepsis survival, ARDS progression, or reducing the duration of MV (148, 149). The potential of aspirin as a primary prevention strategy for mitigating the risk of sepsis or ARDS needs further investigation. In the LIPS-A trial, Redaelli et al. conducted a latent class analysis and hypothesized that hyperinflammatory and non-hyperinflammatory subphenotypes may precede the onset of ARDS and remain recognizable over time. Patients in the high-inflammatory subgroup were found to have a higher likelihood of developing ARDS, a greater need for respiratory support, longer hospitalization, and an increased risk of poor prognosis (150). This highlights the importance of individualized antiplatelet therapy based on subphenotype in SA-ARDS. Myelosuppression and coagulation depletion have made thrombocytopenia a common complication of SA-ARDS (151), and future research will how to rationally apply anticoagulants and antiplatelet agents according to platelet dynamics.

## 10 TCM treatment

The growing emphasis on subphenotypes and personalized treatment in sepsis and ARDS coincides with the TCM theory of “treatment based on syndrome differentiation.” TCM has been widely utilized in the clinical diagnosis and treatment of SA-ARDS through multi-pathway, multi-link and multi-target regulation (152–157), as illustrated in Table 1 (158–166). However, it is regrettable that published clinical studies have not effectively characterized the findings of four diagnostic method nor demonstrated the TCM syndrome patterns with SA-ARDS. Moreover, these studies have not succeeded in correlating physicochemical information with TCM subphenotypes or linking them to specific TCM treatments.

**TABLE 1:** TCM clinical trials.

Treatment	Prescription	Patient	Instructions	Outcomes
Chinese Medicine Monotherapy	Dahuang (Rhubarb)	Sepsis (158)	◆ UT (200,000U, iv, qd) ◆ Rhubarb granules (12 g, po/ig, qd) ◆ For 5 days	After treatment, PCT ↓↓.
		ARDS (159)	◆ Rhubarb leachate (10 g, ig, tid) ◆ For 7 days	On the 5th and 7th, EVLW ↓↓, PVPI ↓↓, P/F ↑↑.
Chinese Medicine Compound	Xuanbai Chengqi decoction (XCD)	ARDS (160)	◆ XCD (400 mL, pr, q12h) ◆ For 3 ∼ 5 days	At 48 and 72 h, Cdyn↑↑, Cst↑↑; at 72 h, PEEP ↓↓; duration of parenteral nutrition ↓↓; complications ↓↓; 28-day mortality ↓↓.
Chinese Medicine Injection	XueBiJing injection (XBJ)	SCAP (161)	◆ XBJ (100 mL, iv, q12h) ◆ For 5 to 7 days	On the 8th, PSI improvement rate ↑↑; 28-day mortality ↓↓; duration of MV ↓↓; ICU stay ↓↓.
		Sepsis (162)	◆ XBJ (100 mL, iv, q12h) ◆ For 5 days	28-day mortality ↓↓; ICU mortality ↓↓; in-hospital mortality ↓↓; ICU stay ↓↓; on the 28th, duration of MV ↓↓; on the 6th, SOFA ↓↓.
	Shenfu injection (SFI)	Septic shock with MV (163)	◆ SFI (50 mL, iv, q12h) ◆ Until discharged or died	28-day mortality ↓↓; duration of IMV ↓↓; duration of vasopressor therapy ↓↓, ICU stay ↓↓; days without organ failure ↓↓; ICU cost ↓↓; hospital cost ↓↓.
	Tanreqing injection (TRQ)	ICU patients with MV (164)	◆ TRQ (iv, at least once) ◆ During ICU.	Duration of IMV ↓↓; ICU mortality ↓↓.
Acupuncture	Zusanli (ST36) and Guanyuan (RN4)	Sepsis (165)	◆ ST36 and RN4 (EA, 30 min/time, bid) ◆ For 7 days.	On the 3rd and 7th, APACHE II ↓↓; on the 7th, CD3 + ↑↑, CD4 + ↑↑, CD8 + ↓↓, CD4 + /CD8 + ↑↑, HLA-DR ↑↑
Qigong	The sitting Baduanjin (SBE)	Sepsis with NIV (166)	◆ SBE (15∼20 min/time, bid) ◆ Until discharged	On the day of transfer out of ICU, muscle strength ↑↑, ADL ↑↑; duration of NIV ↓↓; length of total stay ↓↓; hospital cost ↓↓.

↓↓, significantly reduce; ↑↑, significantly increase; UT, Ulinastatin; SCAP, Critical ill patients with severe community-acquired pneumonia; EVLW, Extravascular lung water; PVPI, pulmonary vascular permeability index; Cdyn, Dynamic Lung Compliance; Cst, Static Lung Compliance; PSI, Pneumonia severity index; EA, Electroacupuncture; HLA-DR, human leukocyte antigen-DR; ADL, activities of daily living.

## 11 Conclusion and future directions

The treatment of SA-ARDS traditionally focuses on resuscitation, anti-infection, and organ support. However, the heterogeneity in response to identical treatments has highlighted the critical importance of subphenotype identification and personalized treatment in the management of SA-ARDS. For example, SA-ARDS can be classified based on their infection origin, such as pulmonary sepsis and extrapulmonary sepsis; according to the causative pathogen, including viral infections, MDRO infections, and mixed viral-bacterial infections; or by the route of infection, there are community-acquired versus hospital-acquired. Immune response states define the hyperinflammatory subphenotype and hypoinflammatory subphenotype. Imaging patterns distinguish the focal lesion subgroup, which typically presents with local consolidation, from the diffuse lesion subgroup, which mainly shows ground-glass opacity. The presence of shock can also help gauge the severity of the disease. There are also subgroups that specifically target biomarkers. These subphenotypes can overlap with each other. Real-time adjustments to therapeutic regimens can be made by dynamically monitoring clinical phenotypes and host-microecological interactions. For example, the hyperinflammatory subphenotype, which is characterized by elevated IL-6 and other inflammatory markers, may benefit more from fluid-restrictive strategies, glucocorticoid and statin anti-inflammatory therapies, high PEEP reperfusion, and tolizumab-targeted therapy. Rapidly matching subphenotypes with more effective treatments enhances the precision and efficiency of the bundle campaign, so as to improve the prognosis of SA-ARDS.

The clinical management of SA-ARDS should progressively move from traditional organ support to precision medicine based on biomarkers and pathomechanisms. In recent years, the combination of multi-omics technologies and artificial intelligence has facilitated the construction of predictive models for identifying high-risk patients, optimizing the treatment plan, and allocating resources. The establishment of an integrated model of “typing-warning-intervention” for SA-ARDS will help to realize the paradigm shift from population-based to individualized.

## 12 Search strategy and selection criteria

We conducted a comprehensive search of the PubMed database from its inception until Oct 30, 2024, using the following search terms: “acute respiratory distress syndrome,” “ARDS,” “acute lung injury,” “ALI,” “sepsis,” “septic-associated acute respiratory distress syndrome,” “sepsis-associated acute lung injury,” “COVID-19,” “SARS-CoV-2.” In addition, we consulted relevant reviews, WHO news reports, and select historically significant articles. Relevant studies were manually screened by reviewing the abstracts and, when necessary, accessing the full articles for further evaluation.

## References

[1] Evans L Rhodes A Alhazzani W Antonelli M Coopersmith C French C Surviving sepsis campaign: International guidelines for management of sepsis and septic shock 2021. *Intensive Care Med.* (2021) 47:1181–247. 10.1007/s00134-021-06506-y 34599691 PMC8486643

[2] Rudd K Johnson S Agesa K Shackelford K Tsoi D Kievlan D Global, regional, and national sepsis incidence and mortality, 1990-2017: Analysis for the Global burden of disease study. *Lancet.* (2020) 395:200–11. 10.1016/S0140-6736(19)32989-7 31954465 PMC6970225

[3] Arefian H Heublein S Scherag A Brunkhorst F Younis M Moerer O Hospital-related cost of sepsis: A systematic review. *J Infection.* (2017) 74:107–17. 10.1016/j.jinf.2016.11.006 27884733

[4] Mayr F Talisa V Balakumar V Chang C Fine M Yende S. Proportion and cost of unplanned 30-day readmissions after sepsis compared with other medical conditions. *JAMA.* (2017) 317:530–1. 10.1001/jama.2016.20468 28114505 PMC13565019

[5] Prescott H Langa K Liu V Escobar G Iwashyna T. Increased 1-year healthcare use in survivors of severe sepsis. *Am J Respir Crit Care Med.* (2014) 190:62–9. 10.1164/rccm.201403-0471OC 24872085 PMC4226030

[6] Iwashyna T Ely E Smith D Langa K. Long-term cognitive impairment and functional disability among survivors of severe sepsis. *JAMA.* (2010) 304:1787–94. 10.1001/jama.2010.1553 20978258 PMC3345288

[7] Reinhart K Daniels R Kissoon N Machado F Schachter R Finfer S. Recognizing sepsis as a global health priority - A WHO resolution. *N Engl J Med.* (2017) 377:414–7. 10.1056/NEJMp1707170 28658587

[8] Sun B Lei M Zhang J Kang H Liu H Zhou F. Acute lung injury caused by sepsis: How does it happen? *Front Med (Lausanne).* (2023) 10:1289194. 10.3389/fmed.2023.1289194 38076268 PMC10702758

[9] Bos L Ware L. Acute respiratory distress syndrome: Causes, pathophysiology, and phenotypes. *Lancet.* (2022) 400:1145–56. 10.1016/S0140-6736(22)01485-4 36070787

[10] Auriemma C Zhuo H Delucchi K Deiss T Liu T Jauregui A Acute respiratory distress syndrome-attributable mortality in critically ill patients with sepsis. *Intensive Care Med.* (2020) 46:1222–31. 10.1007/s00134-020-06010-9 32206845 PMC7224051

[11] Bellani G Laffey J Pham T Fan E Brochard L Esteban A Epidemiology, patterns of care, and mortality for patients with acute respiratory distress syndrome in intensive care units in 50 countries. *JAMA.* (2016) 315:788–800. 10.1001/jama.2016.0291 26903337

[12] Matthay M Arabi Y Arroliga A Bernard G Bersten A Brochard L A new global definition of acute respiratory distress syndrome. *Am J Respir Crit Care Med.* (2024) 209:37–47. 10.1164/rccm.202303-0558WS 37487152 PMC10870872

[13] Grasselli G Calfee C Camporota L Poole D Amato M Antonelli M ESICM guidelines on acute respiratory distress syndrome: Definition, phenotyping and respiratory support strategies. *Intensive Care Med.* (2023) 49:727–59. 10.1007/s00134-023-07050-7 37326646 PMC10354163

[14] Takeuchi O Akira S. Pattern recognition receptors and inflammation. *Cell.* (2010) 140:805–20. 10.1016/j.cell.2010.01.022 20303872

[15] Ni R Jiang L Zhang C Liu M Luo Y Hu Z Biologic mechanisms of macrophage phenotypes responding to infection and the novel therapies to moderate inflammation. *Int J Mol Sci.* (2023) 24:8358. 10.3390/ijms24098358 37176064 PMC10179618

[16] Lewis D Chan D Pinheiro D Armitage-Chan E Garden OA. The immunopathology of sepsis: Pathogen recognition, systemic inflammation, the compensatory anti-inflammatory response, and regulatory T cells. *J Vet Intern Med.* (2012) 26:457–82. 10.1111/j.1939-1676.2012.00905.x 22428780 PMC7166777

[17] Wu M Mi B Liu L Ma H Jiang C Jiang S Genetic polymorphisms, biomarkers and signaling pathways associated with septic shock: From diagnosis to therapeutic targets. *Burns Trauma.* (2024) 12:tkae006. 10.1093/burnst/tkae006 38716051 PMC11074594

[18] Bhatia M Moochhala S. Role of inflammatory mediators in the pathophysiology of acute respiratory distress syndrome. *J Pathol.* (2004) 202:145–56. 10.1002/path.1491 14743496

[19] Qi X Luo Y Xiao M Zhang Q Luo J Ma L Mechanisms of alveolar type 2 epithelial cell death during acute lung injury. *Stem Cells.* (2023) 41:1113–32. 10.1093/stmcls/sxad074 37715783

[20] Vassiliou A Kotanidou A Dimopoulou I Orfanos S. Endothelial damage in acute respiratory distress syndrome. *Int J Mol Sci.* (2020) 21:8793. 10.3390/ijms21228793 33233715 PMC7699909

[21] Qiao X Yin J Zheng Z Li L Feng X. Endothelial cell dynamics in sepsis-induced acute lung injury and acute respiratory distress syndrome: Pathogenesis and therapeutic implications. *Cell Commun Signal.* (2024) 22:241. 10.1186/s12964-024-01620-y 38664775 PMC11046830

[22] Guo R Ward P. Role of oxidants in lung injury during sepsis. *Antioxid Redox Signal.* (2007) 9:1991–2002. 10.1089/ars.2007.1785 17760509

[23] Galley H. Oxidative stress and mitochondrial dysfunction in sepsis. *Br J Anaesth.* (2011) 107:57–64. 10.1093/bja/aer093 21596843

[24] Girardis M David S Ferrer R Helms J Juffermans N Martin-Loeches I Understanding, assessing and treating immune, endothelial and haemostasis dysfunctions in bacterial sepsis. *Intensive Care Med.* (2024) 50:1580–92. 10.1007/s00134-024-07586-2 39222142

[25] Pfeiler S Stark K Massberg S Engelmann B. Propagation of thrombosis by neutrophils and extracellular nucleosome networks. *Haematologica.* (2017) 102:206–13. 10.3324/haematol.2016.142471 27927771 PMC5286929

[26] Gaertner F Massberg S. Blood coagulation in immunothrombosis—At the frontline of intravascular immunity. *Semin Immunol.* (2016) 28:561–9. 10.1016/j.smim.2016.10.010 27866916

[27] Opal S Esmon C. Bench-to-bedside review: Functional relationships between coagulation and the innate immune response and their respective roles in the pathogenesis of sepsis. *Crit Care.* (2003) 7:23–38. 10.1186/cc1854 12617738 PMC154114

[28] Toner P McAuley D Shyamsundar M. Aspirin as a potential treatment in sepsis or acute respiratory distress syndrome. *Crit Care.* (2015) 19:374. 10.1186/s13054-015-1091-6 26494395 PMC4619098

[29] Rizzo A Haeger S Oshima K Yang Y Wallbank A Jin Y Alveolar epithelial glycocalyx degradation mediates surfactant dysfunction and contributes to acute respiratory distress syndrome. *JCI Insight.* (2022) 7:e154573. 10.1172/jci.insight.154573 34874923 PMC8855818

[30] Menezes S Bozza P Neto H Laranjeira A Negri E Capelozzi V Pulmonary and extrapulmonary acute lung injury: Inflammatory and ultrastructural analyses. *J Appl Physiol.* (2005) 98:1777–83. 10.1152/japplphysiol.01182.2004 15649870

[31] Calfee C Janz D Bernard G May A Kangelaris K Matthay M Distinct molecular phenotypes of direct vs indirect ARDS in single-center and multicenter studies. *Chest.* (2015) 147:1539–48. 10.1378/chest.14-2454 26033126 PMC4451708

[32] Pelosi P D’Onofrio D Chiumello D Paolo S Chiara G Capelozzi V Pulmonary and extrapulmonary acute respiratory distress syndrome are different. *Eur Respiratory J.* (2003) 22:48s–56s. 10.1183/09031936.03.00420803 12946001

[33] Goodman L Fumagalli R Tagliabue P Tagliabue M Ferrario M Gattinoni L Adult respiratory distress syndrome due to pulmonary and extrapulmonary causes: CT, clinical, and functional correlations. *Radiology.* (1999) 213:545–52. 10.1148/radiology.213.2.r99nv42545 10551239

[34] Gattinoni L Pelosi P Suter P Pedoto A Vercesi P Lissoni A. Acute respiratory distress syndrome caused by pulmonary and extrapulmonary disease. Different syndromes? *Am J Respir Crit Care Med.* (1998) 158:3–11. 10.1164/ajrccm.158.1.9708031 9655699

[35] Morisawa K Fujitani S Taira Y Kushimoto S Kitazawa Y Okuchi K Difference in pulmonary permeability between indirect and direct acute respiratory distress syndrome assessed by the transpulmonary thermodilution technique: A prospective, observational, multi-institutional study. *J Intensive Care.* (2014) 2:24. 10.1186/2052-0492-2-24 25520836 PMC4267584

[36] Agarwal R Srinivas R Nath A Jindal S. Is the mortality higher in the pulmonary vs the extrapulmonary ARDS? A meta analysis. *Chest.* (2008) 133:1463–73. 10.1378/chest.07-2182 17989150

[37] Levy M Evans L Rhodes A. The surviving sepsis campaign bundle: 2018 update. *Crit Care Med.* (2018) 46:997–1000. 10.1097/CCM.0000000000003119 29767636

[38] Marik P Linde-Zwirble W Bittner E Sahatjian J Hansell D. Fluid administration in severe sepsis and septic shock, patterns and outcomes: An analysis of a large national database. *Intensive Care Med.* (2017) 43:625–32. 10.1007/s00134-016-4675-y 28130687

[39] Staub N. Pulmonary edema: Physiologic approaches to management. *Chest.* (1978) 74:559–64. 10.1378/chest.74.5.559 738094

[40] Sakr Y Vincent J Reinhart K Groeneveld J Michalopoulos A Sprung C High tidal volume and positive fluid balance are associated with worse outcome in acute lung injury. *Chest.* (2005) 128:3098–108. 10.1378/chest.128.5.3098 16304249

[41] van Mourik N Metske H Hofstra J Binnekade J Geerts B Schultz M Cumulative fluid balance predicts mortality and increases time on mechanical ventilation in ARDS patients: An observational cohort study. *PLoS One.* (2019) 14:e0224563. 10.1371/journal.pone.0224563 31665179 PMC6821102

[42] Ingelse S IJland M van Loon L Bem R van Woensel J Lemson J. Early restrictive fluid resuscitation has no clinical advantage in experimental severe pediatric acute respiratory distress syndrome. *Am J Physiol Lung Cell Mol Physiol.* (2021) 320:L1126–36. 10.1152/ajplung.00613.2020 33826416

[43] Famous K Delucchi K Ware L Kangelaris K Liu K Thompson B Acute respiratory distress syndrome subphenotypes respond differently to randomized fluid management strategy. *Am J Respir Crit Care Med.* (2017) 195:331–8. 10.1164/rccm.201603-0645OC 27513822 PMC5328179

[44] Murphy C Schramm G Doherty J Reichley R Gajic O Afessa B The importance of fluid management in acute lung injury secondary to septic shock. *Chest.* (2009) 136:102–9. 10.1378/chest.08-2706 19318675

[45] Corl K Levy M Holder A Douglas I Linde-Zwirble W Alam A. Moderate IV fluid resuscitation is associated with decreased sepsis mortality. *Crit Care Med.* (2024) 52:e557–67. 10.1097/CCM.0000000000006394 39177437 PMC11469629

[46] Malbrain M Van Regenmortel N Saugel B De Tavernier B Van Gaal P Joannes-Boyau O Principles of fluid management and stewardship in septic shock: It is time to consider the four D’s and the four phases of fluid therapy. *Ann Intensive Care.* (2018) 8:66. 10.1186/s13613-018-0402-x 29789983 PMC5964054

[47] Silversides J Major E Ferguson A Mann E McAuley D Marshall J Conservative fluid management or deresuscitation for patients with sepsis or acute respiratory distress syndrome following the resuscitation phase of critical illness: A systematic review and meta-analysis. *Intensive Care Med.* (2017) 43:155–70. 10.1007/s00134-016-4573-3 27734109

[48] Meyer N Prescott H. Sepsis and septic shock. *N Engl J Med.* (2024) 391:2133–46. 10.1056/NEJMra2403213 39774315

[49] Bakaletz L. Viral–bacterial co-infections in the respiratory tract. *Curr Opin Microbiol.* (2017) 35:30–5. 10.1016/j.mib.2016.11.003 27940028 PMC7108227

[50] Shah N Greenberg J McNulty M Gregg K Riddell J Mangino J Bacterial and viral co-infections complicating severe influenza: Incidence and impact among 507 U.S. patients, 2013-14. *J Clin Virol.* (2016) 80:12–9. 10.1016/j.jcv.2016.04.008 27130980 PMC7185824

[51] Klein E Monteforte B Gupta A Jiang W May L Hsieh Y The frequency of influenza and bacterial coinfection: A systematic review and meta-analysis. *Influenza Other Respir Viruses.* (2016) 10:394–403. 10.1111/irv.12398 27232677 PMC4947938

[52] Bardi T Pintado V Gomez-Rojo M Escudero-Sanchez R Azzam Lopez A Diez-Remesal Y Nosocomial infections associated to COVID-19 in the intensive care unit: Clinical characteristics and outcome. *Eur J Clin Microbiol Infect Dis.* (2021) 40:495–502. 10.1007/s10096-020-04142-w 33389263 PMC7778834

[53] Wu H Chang P Chen K Lin I Hsih W Tsai W Coronavirus disease 2019 (COVID-19) associated bacterial coinfection: Incidence, diagnosis and treatment. *J Microbiol Immunol Infect.* (2022) 55:985–92. 10.1016/j.jmii.2022.09.006 36243668 PMC9536868

[54] Ali Y Lynch N Khatri P Bamigbola I Chan A Yabuki M Secondary complement deficiency impairs anti-microbial immunity to *Klebsiella pneumoniae* and Staphylococcus aureus during severe acute COVID-19. *Front Immunol.* (2022) 13:841759. 10.3389/fimmu.2022.841759 35572551 PMC9094484

[55] Rodríguez A Avilés-Jurado F Díaz E Schuetz P Trefler S Solé-Violán J Procalcitonin (PCT) levels for ruling-out bacterial coinfection in ICU patients with influenza: A CHAID decision-tree analysis. *J Infect.* (2016) 72:143–51. 10.1016/j.jinf.2015.11.007 26702737

[56] Carbonell R Urgelés S Salgado M Rodríguez A Reyes L Fuentes Y Negative predictive value of procalcitonin to rule out bacterial respiratory co-infection in critical covid-19 patients. *J Infect.* (2022) 85:374–81. 10.1016/j.jinf.2022.06.024 35781017 PMC9245395

[57] Taylor S Anderson W Beam K Taylor B Ellerman J Kowalkowski M. The association between antibiotic delay intervals and hospital mortality among patients treated in the emergency department for suspected sepsis*. *Crit Care Med.* (2021) 49:741. 10.1097/CCM.0000000000004863 33591002

[58] Asner S Desgranges F Schrijver I Calandra T. Impact of the timeliness of antibiotic therapy on the outcome of patients with sepsis and septic shock. *J Infection.* (2021) 82:125–34. 10.1016/j.jinf.2021.03.003 33722641

[59] Pak T Young J McKenna C Agan A DelloStritto L Filbin M Risk of misleading conclusions in observational studies of time-to-antibiotics and mortality in suspected sepsis. *Clin Infect Dis.* (2023) 77:1534–43. 10.1093/cid/ciad450 37531612 PMC10686960

[60] Taylor S Kowalkowski M Skewes S Chou S. Real-world implications of updated surviving sepsis campaign antibiotic timing recommendations*. *Crit Care Med.* (2024) 52:1002–6. 10.1097/CCM.0000000000006240 38385751

[61] Chastre J Wolff M Fagon J Chevret S Thomas F Wermert D Comparison of 8 vs 15 days of antibiotic therapy for ventilator-associated pneumonia in adults: A randomized trial. *JAMA.* (2003) 290:2588–98. 10.1001/jama.290.19.2588 14625336

[62] Busch L Kadri S. Antimicrobial treatment duration in sepsis and serious infections. *J Infect Dis.* (2020) 222:S142–55. 10.1093/infdis/jiaa247 32691838 PMC7372214

[63] Prkno A Wacker C Brunkhorst F Schlattmann P. Procalcitonin-guided therapy in intensive care unit patients with severe sepsis and septic shock–A systematic review and meta-analysis. *Crit Care.* (2013) 17:R291. 10.1186/cc13157 24330744 PMC4056085

[64] Wirz Y Meier M Bouadma L Luyt C Wolff M Chastre J Effect of procalcitonin-guided antibiotic treatment on clinical outcomes in intensive care unit patients with infection and sepsis patients: A patient-level meta-analysis of randomized trials. *Crit Care.* (2018) 22:191. 10.1186/s13054-018-2125-7 30111341 PMC6092799

[65] Kyriazopoulou E Liaskou-Antoniou L Adamis G Panagaki A Melachroinopoulos N Drakou E Procalcitonin to reduce long-term infection-associated adverse events in sepsis. A randomized trial. *Am J Respiratory Crit Care Med.* (2021) 203:202–10. 10.1164/rccm.202004-1201OC 32757963 PMC7874409

[66] Chaudhuri D Nei A Rochwerg B Balk R Asehnoune K Cadena R 2024 focused update: Guidelines on use of corticosteroids in sepsis, acute respiratory distress syndrome, and community-acquired pneumonia. *Crit Care Med.* (2024) 52:e219. 10.1097/CCM.0000000000006172 38240492

[67] Tomazini B Maia I Cavalcanti A Berwanger O Rosa R Veiga V Effect of dexamethasone on days alive and ventilator-free in patients with moderate or severe acute respiratory distress syndrome and COVID-19: The CoDEX randomized clinical trial. *JAMA.* (2020) 324:1307–16. 10.1001/jama.2020.17021 32876695 PMC7489411

[68] Guillon A Jouan Y Kassa-Sombo A Paget C Dequin P. Hydrocortisone rapidly and significantly reduces the IL-6 level in blood and lungs of patients with COVID-19-related ARDS. *Crit Care.* (2024) 28:101. 10.1186/s13054-024-04887-2 38549157 PMC10976785

[69] Annane D Renault A Brun-Buisson C Megarbane B Quenot J Siami S Hydrocortisone plus fludrocortisone for adults with septic shock. *N Engl J Med.* (2018) 378:809–18. 10.1056/NEJMoa1705716 29490185

[70] Tongyoo S Permpikul C Mongkolpun W Vattanavanit V Udompanturak S Kocak M Hydrocortisone treatment in early sepsis-associated acute respiratory distress syndrome: Results of a randomized controlled trial. *Crit Care.* (2016) 20:329. 10.1186/s13054-016-1511-2 27741949 PMC5065699

[71] Heming N Renault A Kuperminc E Brun-Buisson C Megarbane B Quenot J Hydrocortisone plus fludrocortisone for community acquired pneumonia-related septic shock: A subgroup analysis of the APROCCHSS phase 3 randomised trial. *Lancet Respir Med.* (2024) 12:366–74. 10.1016/S2213-2600(23)00430-7 38310918

[72] Pirracchio R Venkatesh B Legrand M. Low-dose corticosteroids for critically Ill adults with severe pulmonary infections: A review. *JAMA.* (2024) 332:318–28. 10.1001/jama.2024.6096 38865154 PMC13475641

[73] Battaglini D Iavarone I Al-Husinat L Ball L Robba C Silva P Anti-inflammatory therapies for acute respiratory distress syndrome. *Expert Opin Investig Drugs.* (2023) 32:1143–55. 10.1080/13543784.2023.2288080 37996088

[74] Radbakhsh S Katsiki N Santos R Mikhailidis D Mantzoros C Sahebkar A. Effects of statins on specialized pro-resolving mediators: An additional pathway leading to resolution of inflammation. *Metabolism.* (2022) 132:155211. 10.1016/j.metabol.2022.155211 35533891

[75] He Y Li X Gasevic D Brunt E McLachlan F Millenson M Statins and multiple noncardiovascular outcomes. *Ann Int Med.* (2018) 169:543–53. 10.7326/M18-0808 30304368

[76] Hofmaenner D Kleyman A Press A Bauer M Singer M. The many roles of cholesterol in sepsis: A review. *Am J Respir Crit Care Med.* (2022) 205:388–96. 10.1164/rccm.202105-1197TR 34715007 PMC8886946

[77] Terblanche M Pinto R Whiteley C Brett S Beale R Adhikari N. Statins do not prevent acute organ failure in ventilated ICU patients: Single-centre retrospective cohort study. *Crit Care.* (2011) 15:R74. 10.1186/cc10063 21356051 PMC3222007

[78] Pertzov B Eliakim-Raz N Atamna H Trestioreanu A Yahav D Leibovici L. Hydroxymethylglutaryl-CoA reductase inhibitors (statins) for the treatment of sepsis in adults - A systematic review and meta-analysis. *Clin Microbiol Infect.* (2019) 25:280–9. 10.1016/j.cmi.2018.11.003 30472427

[79] Lee C Lee M Hsu T Porta L Chang S Yo C A population-based cohort study on the drug-specific effect of statins on sepsis outcome. *Chest.* (2018) 153:805–15. 10.1016/j.chest.2017.09.024 28962887

[80] Hashem M Hopkins R Colantuoni E Dinglas V Sinha P Aronson Friedman L Six-month and 12-month patient outcomes based on inflammatory subphenotypes in sepsis-associated ARDS: Secondary analysis of SAILS-ALTOS trial. *Thorax.* (2022) 77:22–30. 10.1136/thoraxjnl-2020-216613 34112703 PMC8660939

[81] Boyle A Ferris P Bradbury I Conlon J Shankar-Hari M Rogers A Baseline plasma IL-18 may predict simvastatin treatment response in patients with ARDS: A secondary analysis of the HARP-2 randomised clinical trial. *Crit Care.* (2022) 26:164. 10.1186/s13054-022-04025-w 35672834 PMC9175337

[82] Rogers A Guan J Trtchounian A Hunninghake G Kaimal R Desai M Association of elevated plasma interleukin-18 level with increased mortality in a clinical trial of statin treatment for acute respiratory distress syndrome. *Crit Care Med.* (2019) 47:1089–96. 10.1097/CCM.0000000000003816 31206358 PMC6629502

[83] Wang S Xie X Lei T Zhang K Lai B Zhang Z Statins attenuate activation of the NLRP3 inflammasome by oxidized LDL or TNFα in vascular endothelial cells through a PXR-dependent mechanism. *Mol Pharmacol.* (2017) 92:256–64. 10.1124/mol.116.108100 28546421

[84] Zhang K Liu W Liang H. Effect of statins on sepsis and inflammatory factors: A Mendelian randomization study. *Eur J Clin Invest.* (2024) 54:e14164. 10.1111/eci.14164 38229409

[85] Wu Y Wang L Li Y Cao Y Wang M Deng Z Immunotherapy in the context of sepsis-induced immunological dysregulation. *Front Immunol.* (2024) 15:1391395. 10.3389/fimmu.2024.1391395 38835773 PMC11148279

[86] Toniati P Piva S Cattalini M Garrafa E Regola F Castelli F Tocilizumab for the treatment of severe COVID-19 pneumonia with hyperinflammatory syndrome and acute respiratory failure: A single center study of 100 patients in Brescia. *Italy. Autoimmun Rev.* (2020) 19:102568. 10.1016/j.autrev.2020.102568 32376398 PMC7252115

[87] Menzella F Fontana M Salvarani C Massari M Ruggiero P Scelfo C Efficacy of tocilizumab in patients with COVID-19 ARDS undergoing noninvasive ventilation. *Critical Care.* (2020) 24:589. 10.1186/s13054-020-03306-6 32993751 PMC7523258

[88] Rambaldi A Gritti G Micò MC Frigeni M Borleri G Salvi A. Endothelial injury and thrombotic microangiopathy in COVID-19: Treatment with the lectin-pathway inhibitor narsoplimab. *Immunobiology.* (2020) 225:152001. 10.1016/j.imbio.2020.152001 32943233 PMC7415163

[89] Mannes M Schmidt C Nilsson B Ekdahl K Huber-Lang M. Complement as driver of systemic inflammation and organ failure in trauma, burn, and sepsis. *Semin Immunopathol.* (2021) 43:773–88. 10.1007/s00281-021-00872-x 34191093 PMC8243057

[90] Annane D Heming N Grimaldi-Bensouda L Frémeaux-Bacchi V Vigan M Roux A Eculizumab as an emergency treatment for adult patients with severe COVID-19 in the intensive care unit: A proof-of-concept study. *EClinicalMedicine.* (2020) 28:100590. 10.1016/j.eclinm.2020.100590 33173853 PMC7644240

[91] Lynch N Chan A Ali Y Khatri P Bamigbola I Demopulos G Inhibition of the lectin pathway of complement ameliorates hypocomplementemia and restores serum bactericidal activity in patients with severe COVID-19. *Clin Transl Med.* (2022) 12:e980. 10.1002/ctm2.980 35839316 PMC9286524

[92] Hutchins N Unsinger J Hotchkiss R Ayala A. The new normal: Immuno-modulatory agents against sepsis immune suppression. *Trends Mol Med.* (2014) 20:224–33. 10.1016/j.molmed.2014.01.002 24485901 PMC3976785

[93] Francois B Jeannet R Daix T Walton A Shotwell M Unsinger J Interleukin-7 restores lymphocytes in septic shock: The IRIS-7 randomized clinical trial. *JCI Insight.* (2018) 3:e98960. 10.1172/jci.insight.98960 29515037 PMC5922293

[94] Daix T Mathonnet A Brakenridge S Dequin P Mira J Berbille F Intravenously administered interleukin-7 to reverse lymphopenia in patients with septic shock: A double-blind, randomized, placebo-controlled trial. *Ann Intensive Care.* (2023) 13:17. 10.1186/s13613-023-01109-w 36906875 PMC10008152

[95] Hiruma T Tsuyuzaki H Uchida K Trapnell B Yamamura Y Kusakabe Y IFN-β improves sepsis-related alveolar macrophage dysfunction and postseptic acute respiratory distress syndrome-related mortality. *Am J Respir Cell Mol Biol.* (2018) 59:45–55. 10.1165/rcmb.2017-0261OC 29365277 PMC6835072

[96] Sekheri M Rizo-Téllez S Othman A El Kebir D Filep J. Interferon-β regulates proresolving lipids to promote the resolution of acute airway inflammation. *Proc Natl Acad Sci U S A.* (2022) 119:e2201146119. 10.1073/pnas.2201146119 35878041 PMC9351544

[97] Bellingan G Maksimow M Howell D Stotz M Beale R Beatty M The effect of intravenous interferon-beta-1a (FP-1201) on lung CD73 expression and on acute respiratory distress syndrome mortality: An open-label study. *Lancet Respir Med.* (2014) 2:98–107. 10.1016/S2213-2600(13)70259-5 24503265

[98] Wang Z Zhang W Chen L Lu X Tu Y. Lymphopenia in sepsis: A narrative review. *Crit Care.* (2024) 28:315. 10.1186/s13054-024-05099-4 39304908 PMC11414153

[99] Zhang Y Zhou Y Lou J Li J Bo L Zhu K PD-L1 blockade improves survival in experimental sepsis by inhibiting lymphocyte apoptosis and reversing monocyte dysfunction. *Crit Care.* (2010) 14:R220. 10.1186/cc9354 21118528 PMC3220038

[100] Chang K Burnham C Compton S Rasche D Mazuski R McDonough J Blockade of the negative co-stimulatory molecules PD-1 and CTLA-4 improves survival in primary and secondary fungal sepsis. *Crit Care.* (2013) 17:R85. 10.1186/cc12711 23663657 PMC3706819

[101] Chang K Svabek C Vazquez-Guillamet C Sato B Rasche D Wilson S Targeting the programmed cell death 1: Programmed cell death ligand 1 pathway reverses T cell exhaustion in patients with sepsis. *Crit Care.* (2014) 18:R3. 10.1186/cc13176 24387680 PMC4056005

[102] Hotchkiss R Colston E Yende S Angus D Moldawer L Crouser. Immune checkpoint inhibition in sepsis: A phase 1b randomized, placebo-controlled, single ascending dose study of antiprogrammed cell death-ligand 1 antibody (BMS-936559). *Crit Care Med.* (2019) 47:632–42. 10.1097/CCM.0000000000003685 30747773 PMC7254685

[103] Hotchkiss R Colston E Yende S Crouser, Martin G Albertson T Immune checkpoint inhibition in sepsis: A Phase 1b randomized study to evaluate the safety, tolerability, pharmacokinetics, and pharmacodynamics of nivolumab. *Intensive Care Med.* (2019) 45:1360–71. 10.1007/s00134-019-05704-z 31576433 PMC9006384

[104] Watanabe E Nishida O Kakihana Y Odani M Okamura T Harada T Pharmacokinetics, pharmacodynamics, and safety of nivolumab in patients with sepsis-induced immunosuppression: A multicenter, open-label phase 1/2 study. *Shock.* (2020) 53:686–94. 10.1097/SHK.0000000000001443 31513050 PMC7448837

[105] Gillis A Beil M Halevi-Tobias K van Heerden P Sviri S Agur Z. Alleviation of exhaustion-induced immunosuppression and sepsis by immune checkpoint blockers sequentially administered with antibiotics-analysis of a new mathematical model. *Intensive Care Med Exp.* (2019) 7:32. 10.1186/s40635-019-0260-3 31187301 PMC6560115

[106] Gillis A Ben Yaacov A Agur Z. A new method for optimizing sepsis therapy by nivolumab and meropenem combination: importance of early intervention and CTL reinvigoration rate as a response marker. *Front Immunol.* (2021) 12:616881. 10.3389/fimmu.2021.616881 33732241 PMC7959825

[107] Grieco D Maggiore S Roca O Spinelli E Patel B Thille A Non-invasive ventilatory support and high-flow nasal oxygen as first-line treatment of acute hypoxemic respiratory failure and ARDS. *Intensive Care Med.* (2021) 47:851–66. 10.1007/s00134-021-06459-2 34232336 PMC8261815

[108] Perkins G Ji C Connolly B Couper K Lall R Baillie J Effect of noninvasive respiratory strategies on intubation or mortality among patients with acute hypoxemic respiratory failure and COVID-19: The RECOVERY-RS randomized clinical trial. *JAMA.* (2022) 327:546–58. 10.1001/jama.2022.0028 35072713 PMC8787685

[109] Rochwerg B Granton D Wang D Helviz Y Einav S Frat J High flow nasal cannula compared with conventional oxygen therapy for acute hypoxemic respiratory failure: A systematic review and meta-analysis. *Intensive Care Med.* (2019) 45:563–72. 10.1007/s00134-019-05590-5 30888444

[110] Ospina-Tascón G Calderón-Tapia L García A Zarama V Gómez-Álvarez F Álvarez-Saa T Effect of high-flow oxygen therapy vs conventional oxygen therapy on invasive mechanical ventilation and clinical recovery in patients with severe COVID-19: A randomized clinical trial. *JAMA.* (2021) 326:2161–71. 10.1001/jama.2021.20714 34874419 PMC8652598

[111] Frat J Quenot J Badie J Coudroy R Guitton C Ehrmann S Effect of high-flow nasal cannula oxygen vs standard oxygen therapy on mortality in patients with respiratory failure due to Covid-19: The SOHO-COVID randomized clinical trial. *JAMA.* (2022) 328:1212–22. 10.1001/jama.2022.15613 36166027 PMC9516287

[112] Nishimura M. High-flow nasal cannula oxygen therapy in adults. *J Intensive Care.* (2015) 3:15. 10.1186/s40560-015-0084-5 25866645 PMC4393594

[113] Zhao H Wang H Sun F Lyu S An Y. High-flow nasal cannula oxygen therapy is superior to conventional oxygen therapy but not to noninvasive mechanical ventilation on intubation rate: A systematic review and meta-analysis. *Crit Care.* (2017) 21:184. 10.1186/s13054-017-1760-8 28701227 PMC5508784

[114] Ni Y Luo J Yu H Liu D Ni Z Cheng J Can high-flow nasal cannula reduce the rate of endotracheal intubation in adult patients with acute respiratory failure compared with conventional oxygen therapy and noninvasive positive pressure ventilation?: A systematic review and meta-analysis. *Chest.* (2017) 151:764–75. 10.1016/j.chest.2017.01.004 28089816

[115] Grieco D Menga L Cesarano M Rosà T Spadaro S Bitondo M Effect of helmet noninvasive ventilation vs high-flow nasal oxygen on days free of respiratory support in patients with COVID-19 and moderate to severe hypoxemic respiratory failure: The henivot randomized clinical trial. *JAMA.* (2021) 325:1731–43. 10.1001/jama.2021.4682 33764378 PMC7995134

[116] Shen Y Zhang W. High-flow nasal cannula versus noninvasive positive pressure ventilation in acute respiratory failure: Interaction between PaO2/FiO2 and tidal volume. *Crit Care.* (2017) 21:285. 10.1186/s13054-017-1861-4 29166943 PMC5700684

[117] Ovtcharenko N Ho E Alhazzani W Cortegiani A Ergan B Scala R High-flow nasal cannula versus non-invasive ventilation for acute hypercapnic respiratory failure in adults: A systematic review and meta-analysis of randomized trials. *Crit Care.* (2022) 26:348. 10.1186/s13054-022-04218-3 36352457 PMC9648030

[118] Tan D Wang B Cao P Wang Y Sun J Geng P High flow nasal cannula oxygen therapy versus non-invasive ventilation for acute exacerbations of chronic obstructive pulmonary disease with acute-moderate hypercapnic respiratory failure: A randomized controlled non-inferiority trial. *Crit Care.* (2024) 28:250. 10.1186/s13054-024-05040-9 39026242 PMC11264824

[119] Ai T Zhang Z Tan Z Shi Z Li H Zhang S Modified respiratory rate oxygenation index: An early warning index for the need of intubation in COVID-19 patients with high-flow nasal cannula therapy. *J Emerg Med.* (2023) 65:e93–100. 10.1016/j.jemermed.2023.04.026 37479639 PMC10212589

[120] Yau C Lee D Vasudevan A Goh K Wong E Ho A Performance of the ROX index in predicting high flow nasal cannula failure in COVID-19 patients: A systematic review and meta-analysis. *Crit Care.* (2023) 27:320. 10.1186/s13054-023-04567-7 37605238 PMC10441756

[121] Gattarello S Coppola S Chiodaroli E Pozzi T Camporota L Saager L Mechanical Power ratio and respiratory treatment escalation in COVID-19 pneumonia: A secondary analysis of a prospectively enrolled cohort. *Anesthesiology.* (2023) 138:289–98. 10.1097/ALN.0000000000004465 36571571 PMC9904389

[122] Maia G Martins C Marques V Christovam S Prado I Moraes B Derivation and external validation of predictive models for invasive mechanical ventilation in intensive care unit patients with COVID-19. *Ann Intensive Care.* (2024) 14:129. 10.1186/s13613-024-01357-4 39167241 PMC11339005

[123] Girault C Bubenheim M Boyer D Declercq P Schnell G Gouin P ROX index performance to predict high-flow nasal oxygen outcome in Covid-19 related hypoxemic acute respiratory failure. *Ann Intensive Care.* (2024) 14:13. 10.1186/s13613-023-01226-6 38236356 PMC10796865

[124] Karageorgos V Proklou A Vaporidi K. Lung and diaphragm protective ventilation: A synthesis of recent data. *Expert Rev Respir Med.* (2022) 16:375–90. 10.1080/17476348.2022.2060824 35354361

[125] Acute Respiratory Distress Syndrome Network, Brower RG Matthay MA Morris A Schoenfeld D Thompson BT Ventilation with lower tidal volumes as compared with traditional tidal volumes for acute lung injury and the acute respiratory distress syndrome. *N Engl J Med.* (2000) 342:1301–8. 10.1056/NEJM200005043421801 10793162

[126] Brower R Lanken P MacIntyre N Matthay M Morris A Ancukiewicz M Higher versus lower positive end-expiratory pressures in patients with the acute respiratory distress syndrome. *N Engl J Med.* (2004) 351:327–36. 10.1056/NEJMoa032193 15269312

[127] Meade M Cook D Guyatt G Slutsky A Arabi Y Cooper D Ventilation strategy using low tidal volumes, recruitment maneuvers, and high positive end-expiratory pressure for acute lung injury and acute respiratory distress syndrome: A randomized controlled trial. *JAMA.* (2008) 299:637. 10.1001/jama.299.6.637 18270352

[128] Mercat A Richard J Vielle B Jaber S Osman D Diehl J Positive end-expiratory pressure setting in adults with acute lung injury and acute respiratory distress syndrome: A randomized controlled trial. *JAMA.* (2008) 299:646–55. 10.1001/jama.299.6.646 18270353

[129] Briel M Meade M Mercat A Brower R Talmor D Walter S Higher vs lower positive end-expiratory pressure in patients with acute lung injury and acute respiratory distress syndrome: Systematic review and meta-analysis. *JAMA.* (2010) 303:865–73. 10.1001/jama.2010.218 20197533

[130] Siuba M Bulgarelli L Duggal A Cavalcanti A Zampieri F Rey D Differential effect of positive end-expiratory pressure strategies in patients with ARDS: A bayesian analysis of clinical subphenotypes. *Chest.* (2024) 166:754–64. 10.1016/j.chest.2024.04.011 38768777 PMC11489450

[131] Maddali M Churpek M Pham T Rezoagli E Zhuo H Zhao W Validation and utility of ARDS subphenotypes identified by machine-learning models using clinical data: An observational, multicohort, retrospective analysis. *Lancet Respir Med.* (2022) 10:367–77. 10.1016/S2213-2600(21)00461-6 35026177 PMC8976729

[132] Gattinoni L Collino F Camporota L. Assessing lung recruitability: Does it help with PEEP settings? *Intensive Care Med.* (2024) 50:749–51. 10.1007/s00134-024-07351-5 38536421 PMC11078853

[133] Gattinoni L Caironi P Cressoni M Chiumello D Ranieri V Quintel M Lung recruitment in patients with the acute respiratory distress syndrome. *N Engl J Med.* (2006) 354:1775–86. 10.1056/NEJMoa052052 16641394

[134] Beitler J Sarge T Banner-Goodspeed V Gong M Cook D Novack V Effect of titrating positive end-expiratory pressure (PEEP) with an esophageal pressure-guided strategy vs an empirical high PEEP-Fio2 strategy on death and days free from mechanical ventilation among patients with acute respiratory distress syndrome: A randomized clinical trial. *JAMA.* (2019) 321:846–57. 10.1001/jama.2019.0555 30776290 PMC6439595

[135] Sarge T Baedorf-Kassis E Banner-Goodspeed V Novack V Loring S Gong M Effect of esophageal pressure-guided positive end-expiratory pressure on survival from acute respiratory distress syndrome: A Risk-based and mechanistic reanalysis of the EPVent-2 trial. *Am J Respir Crit Care Med.* (2021) 204:1153–63. 10.1164/rccm.202009-3539OC 34464237 PMC8759303

[136] Puybasset L Gusman P Muller J Cluzel P Coriat P Rouby J. Regional distribution of gas and tissue in acute respiratory distress syndrome. III. Consequences for the effects of positive end-expiratory pressure. CT scan ARDS study group. Adult respiratory distress syndrome. *Intensive Care Med.* (2000) 26:1215–27. 10.1007/s001340051340 11089745

[137] Mousa A Klompmaker P Tuinman P. Setting positive end-expiratory pressure: Lung and diaphragm ultrasound. *Curr Opin Crit Care.* (2023) 30:53. 10.1097/MCC.0000000000001119 38085883 PMC10962429

[138] Franchineau G Bréchot N Lebreton G Hekimian G Nieszkowska A Trouillet J Bedside contribution of electrical impedance tomography to setting positive end-expiratory pressure for extracorporeal membrane oxygenation-treated patients with severe acute respiratory distress syndrome. *Am J Respir Crit Care Med.* (2017) 196:447–57. 10.1164/rccm.201605-1055OC 28103448

[139] McNicholas B Ehrmann S Laffey J. Awake prone positioning. *Intensive Care Med.* (2022) 48:1793–5. 10.1007/s00134-022-06893-w 36151334 PMC9510305

[140] Cornejo R Díaz J Tobar E Bruhn A Ramos C González R Effects of prone positioning on lung protection in patients with acute respiratory distress syndrome. *Am J Respir Crit Care Med.* (2013) 188:440–8. 10.1164/rccm.201207-1279OC 23348974

[141] Guérin C Reignier J Richard J Beuret P Gacouin A Boulain T Prone positioning in severe acute respiratory distress syndrome. *N Engl J Med.* (2013) 368:2159–68. 10.1056/NEJMoa1214103 23688302

[142] Yan Y Geng B Liang J Wen Y Bao J Zhong X A prediction model for nonresponsive outcomes in critically ill patients with acute respiratory distress syndrome undergoing prone position ventilation: A retrospective cohort study. *Intensive Crit Care Nurs.* (2025) 86:103804. 10.1016/j.iccn.2024.103804 39180911

[143] Dixon B Smith R Campbell D Moran J Doig G Rechnitzer T Nebulised heparin for patients with or at risk of acute respiratory distress syndrome: A multicentre, randomised, double-blind, placebo-controlled phase 3 trial. *Lancet Respir Med.* (2021) 9:360–72. 10.1016/S2213-2600(20)30470-7 33493448 PMC7826120

[144] Zou Z Huang J Luan Y Yang Z Zhou Z Zhang J Early prophylactic anticoagulation with heparin alleviates mortality in critically ill patients with sepsis: A retrospective analysis from the MIMIC-IV database. *Burns Trauma.* (2022) 10:tkac029. 10.1093/burnst/tkac029 36168402 PMC9501718

[145] Hamid U Krasnodembskaya A Fitzgerald M Shyamsundar M Kissenpfennig A Scott C Aspirin reduces lipopolysaccharide-induced pulmonary inflammation in human models of ARDS. *Thorax.* (2017) 72:971–80. 10.1136/thoraxjnl-2016-208571 28082531 PMC5858553

[146] Trauer J Muhi S McBryde E Harbi S Arabi Y Boyle A Quantifying the effects of prior acetyl-salicylic acid on sepsis-related deaths: An individual patient data meta-analysis using propensity matching. *Crit Care Med.* (2017) 45:1871. 10.1097/CCM.0000000000002654 28799949 PMC5640482

[147] Boyle A Di Gangi S Hamid U Mottram L McNamee L White G Aspirin therapy in patients with acute respiratory distress syndrome (ARDS) is associated with reduced intensive care unit mortality: A prospective analysis. *Crit Care.* (2015) 19:109. 10.1186/s13054-015-0846-4 25887566 PMC4371625

[148] Eisen D Leder K Woods R Lockery J McGuinness S Wolfe R Effect of aspirin on deaths associated with sepsis in healthy older people (ANTISEPSIS): A randomised, double-blind, placebo-controlled primary prevention trial. *Lancet Respir Med.* (2021) 9:186–95. 10.1016/S2213-2600(20)30411-2 32950072 PMC7957956

[149] Kor D Carter R Park P Festic E Banner-Goodspeed V Hinds R Effect of aspirin on development of ARDS in at-risk patients presenting to the emergency department: The LIPS-A randomized clinical trial. *JAMA.* (2016) 315:2406–14. 10.1001/jama.2016.6330 27179988 PMC5450939

[150] Redaelli S von Wedel D Fosset M Suleiman A Chen G Alingrin J Inflammatory subphenotypes in patients at risk of ARDS: Evidence from the LIPS-A trial. *Intensive Care Med.* (2023) 49:1499–507. 10.1007/s00134-023-07244-z 37906258

[151] Leone M Nielsen N Russell L. Ten tips on sepsis-induced thrombocytopenia. *Intensive Care Med.* (2024) 50:1157–60. 10.1007/s00134-024-07478-5 38739278

[152] Xie P Yan L Zhou H Cao H Zheng Y Lu Z Emodin protects against lipopolysaccharide-induced acute lung injury via the JNK/Nur77/c-jun signaling pathway. *Front Pharmacol.* (2022) 13:717271. 10.3389/fphar.2022.717271 35370650 PMC8968870

[153] Zhu H Wang S Shan C Li X Tan B Chen Q Mechanism of protective effect of xuan-bai-cheng-qi decoction on LPS-induced acute lung injury based on an integrated network pharmacology and RNA-sequencing approach. *Respir Res.* (2021) 22:188. 10.1186/s12931-021-01781-1 34183011 PMC8237774

[154] Zou F Zou J Du Q Liu L Li D Zhao L XueBiJing injection improves the symptoms of sepsis-induced acute lung injury by mitigating oxidative stress and ferroptosis. *J Ethnopharmacol.* (2024) 337:118732. 10.1016/j.jep.2024.118732 39181287

[155] Zhang L Zi Y Zhang Y Pei H Zheng X Xie J Shenfu Injection alleviates sepsis-associated lung injury by regulating HIF-1α. *China J Chinese Materia Med.* (2023) 48:6492–9. 10.19540/j.cnki.cjcmm.20230814.701 38212006

[156] Tang B Xie L Wang Y Shi Y Kan W Feng B Exploratory research on the effective chemical basis of Tanreqing injection for treating acute lung injury: In vivo, in vitro and in silico. *J Ethnopharmacol.* (2024) 337:118780. 10.1016/j.jep.2024.118780 39260706

[157] Shi J Piao M Liu C Yang J Guan X Liu H Electroacupuncture pretreatment maintains mitochondrial quality control via HO-1/MIC60 signaling pathway to alleviate endotoxin-induced acute lung injury. *Biochim Biophys Acta Mol Basis Dis.* (2024) 1870:167480. 10.1016/j.bbadis.2024.167480 39209235

[158] Meng F Du C Zhang Y Wang S Zhou Q Wu L Protective effect of rhubarb combined with ulinastatin for patients with sepsis. *Medicine (Baltimore).* (2020) 99:e18895. 10.1097/MD.0000000000018895 32049789 PMC7035124

[159] He J Si X Ji M Huang J Zheng W Wang J Effect of rhubarb on extravascular lung water in patients with acute respiratory distress syndrome. *Rev Assoc Med Bras.* (2017) 63:435–40. 10.1590/1806-9282.63.05.435 28724041

[160] Mao Z Wang H. Effects of Xuanbai Chengqi decoction on lung compliance for patients with exogenous pulmonary acute respiratory distress syndrome. *Drug Des Devel Ther.* (2016) 10:793–8. 10.2147/DDDT.S93165 26929604 PMC4767063

[161] Song Y Yao C Yao Y Han H Zhao X Yu K XueBiJing injection versus placebo for critically ill patients with severe community-acquired pneumonia: A randomized controlled trial. *Crit Care Med.* (2019) 47:e735–43. 10.1097/CCM.0000000000003842 31162191 PMC6727951

[162] Liu S Yao C Xie J Liu H Wang H Lin Z Effect of an herbal-based injection on 28-day mortality in patients with sepsis: The EXIT-SEP randomized clinical trial. *JAMA Intern Med.* (2023) 183:647–55. 10.1001/jamainternmed.2023.0780 37126332 PMC10152378

[163] Li X Huang F Zhu L Luo T Zhang Y Gu H Effects of combination therapy with Shenfu injection in critically ill patients with septic shock receiving mechanical ventilation: A multicentric, real-world study. *Front Pharmacol.* (2022) 13:1041326. 10.3389/fphar.2022.1041326 36438846 PMC9682251

[164] Wang W He Q Wang M Xu J Ji P Zhang R Effects of tanreqing injection on ICU mortality among ICU patients receiving mechanical ventilation: Time-dependent cox regression analysis of a large registry. *Chin J Integr Med.* (2023) 29:782–90. 10.1007/s11655-023-3634-z 36973530

[165] Yang G Hu R Deng A Huang Y Li J. Effects of electro-acupuncture at Zusanli, Guanyuan for sepsis patients and its mechanism through immune regulation. *Chin J Integr Med.* (2016) 22:219–24. 10.1007/s11655-016-2462-9 26825083

[166] Chen M Wang F Huang L Qi T Guo H Zeng R Effect of Sitting Baduanjin exercise on early rehabilitation of sepsis patients with non-invasive ventilation: A randomized controlled trial. *BMC Complement Med Ther.* (2024) 24:330. 10.1186/s12906-024-04626-8 39243078 PMC11378565

